# Sex Work and Parenthood: The Experiences of Female Sex Workers Who Are also Parents or Caregivers: A Scoping Review

**DOI:** 10.3390/ijerph21070852

**Published:** 2024-06-29

**Authors:** Mokhwelepa Leshata Winter, Sumbane Gsakani Olivia

**Affiliations:** School of Medicine, Faculty of Health Science, University of Limpopo, Private Bag X 1106, Sovenga, Polokwane 0727, South Africa; gsakani.sumbane@ul.ac.za

**Keywords:** sex work, parenthood, caregiving, stigma, mental health, financial pressure, criminalization, coping strategies

## Abstract

Complex interactions exist between sex work and parenthood, impacting the lives of those in sex work who also provide care for others. This scoping review aims to review the experiences and challenges of female sex workers who are parents or caregivers, highlighting the socioeconomic, psychological, and legal dimensions of their dual roles. The rationale for this review stems from the recognition that sex workers who are also parents face unique and multifaceted challenges that significantly impact their wellbeing and that of their children. The five stages of a scoping review suggested by Arksey and O’Malley were followed in this study. A comprehensive literature search was conducted across electronic databases such as PubMed, PsycINFO, and Google Scholar. This study covered publications written in English from 2010 to 2023. Studies were selected based on their focus on sex workers who are parents or caregivers. Both qualitative and quantitative research articles were included. Thematic analysis was employed to synthesize findings across the selected studies. Studies published prior to 2013, studies that were not published in English, and studies that did not address the experiences of female sex workers who are parents or caregivers were excluded from this study. The review identified 14 studies meeting the inclusion criteria. Five key themes emerged from this study: (1) social stigma and healthcare discrimination; (2) legal challenges; (3) mental nexus; (4) risk behaviors and exposing children to a hazardous environment; and (5) social support. Sex workers who are also parents or caregivers navigate a multifaceted landscape of challenges and resilience. Policy reforms are needed to reduce stigma, provide financial support, and ensure legal protections for this key population.

## 1. Introduction

Women who work in the sex industry are frequently mothers. These women are frequently considered vectors of diseases, high-risk individuals, or victims who are forced into sex work. Female sex workers are multifaceted individuals with complex lives, and this perspective fails to acknowledge this [1]. Female sex workers frequently experience poverty, violence, marginalization, and psychological distress [1]. These factors have also been found to affect parental bonds. The desire for money, which is frequently derived from meeting the demands of one’s children, is the primary motivator and driver for many female sex workers, according to the literature [2,3,4]. Research conducted in various international contexts has revealed that a significant number of female sex workers who are also parents or caregivers highlight the importance of their children in their lives and attribute their motivation for engaging in sex work to the need to provide for them [5,6]. However, little is known about how parenthood affects behavior, HIV risk, and other healthcare outcomes for female sex workers, as well as how they become mothers [7].

Mixed findings have been discovered in a modest corpus of mostly qualitative studies on the connection between sexual risk and motherhood. Both increased risk (e.g., accepting more money for condom-free sex in order to satisfy an urgent child’s need) and decreased risk (e.g., having children encourages increased condom use and decreased drug use) have been linked to motherhood [8,9,10,11,12]. However, the criminalization of sex work has also resulted in a number of violations of human rights and health, including harm to the familial bonds of sex workers and an obstruction to their capacity to parent [13]. That being said, not much is known about the difficulties faced by sex workers who are expecting or who are parenting [14]. It is interesting to note that some researchers have hypothesized a close connection between sex work and motherhood. Studies of sex workers in developing nations have shown high rates of pregnancy and dependent childbirth, with up to 90% of sex workers having children [13]. Furthermore, several qualitative researchers have suggested that many women pursue careers in sex work in order to provide for their families [10]. This is especially true in Canada, where studies have shown that sex work was one of the few economically feasible ways to support the families of indoor sex workers, especially for low-income women and migrant workers who spoke little or no English [15]. American academics have shown that, unlike popular belief, sex workers are deeply committed to raising their children [10].

While some reports from sex workers highlight the flexibility, increased income, and financial independence from intimate partners as advantages of working while being a mother, there are also many drawbacks, such as the risk of contracting STIs, aggression, and stigma [10,13,15]. Researchers using qualitative methods have shown that stigma is pervasive among those who work in the sex industry and has been connected to stress, sadness, and avoiding medical care [13,16]. Researchers have shown that stigma can cause marginalized sex workers to cut social links with friends and relatives in a number of contexts [13]. Consequently, this could hinder sex workers’ capacity to raise children, mostly because it will result in less access to resources and assistance. It is interesting to note that drug use and drug-related issues, such as uncertainty about the effectiveness of services, fear of reporting to the police, and external local control, were found to be barriers to prenatal care by researchers researching mothers who use drugs [13]. The majority of drug-using sex workers are devoted to providing for their children, despite the difficulties that come with being a parent and using illegal drugs [13,17].

Since many sex workers have given birth, as evidenced by the literature, they identify as mothers, even when authorities, families, and socio-institutional systems do not [18]. Mothers who operate as sex workers face intense societal and governmental scrutiny [18]. Owing to their profession, they are frequently viewed as inherently dangerous individuals who endanger their children from a sociolegal and moral standpoint [18]. Samtani and Trejos-Castillo elucidate that the social criticism of sex work as a job eclipses the function of a mother as a parent, depriving a mother who works in the industry of the opportunity to be fairly assessed for her parenting abilities [19]. For good reason, a lot of parents of sex workers do indeed speak of living in continual fear of Child Protection organizations [17]. More than one-third of the 350 sex workers who are also parents surveyed in Duff’s research from 2014 said their child had been arrested [20]. Although there is a greater chance of child apprehension for all prostitutes, street-based prostitutes—that is, those who solicit customers from public places such as parks, alleys, and street corners—face a risk of child apprehension that can be up to 2.5 times higher than that of indoor prostitutes [20]. The high apprehension rates among parents of street-based sex workers seem to be associated with a number of intersecting marginalizations that may exacerbate their continued struggles to retain custody of their children [20]. As a result, societal, economic, or health-related obstacles to parenting are encountered by almost all parents who are also street-based sex workers in North America.

It is crucial to remember that the authors looked into research spanning 2010 to 2023. The authors felt it was important to draw attention to how COVID-19 affected parents who work in the sex industry. The global epidemic of COVID-19 has significantly impacted the earnings of sex workers [21]. Many female sex workers are battling with housing and food poverty as a result of social distancing efforts, which have decreased demand for sex workers and resulted in a loss of customers and revenue [21]. Due to financial constraints, many female prostitutes have resorted to dangerous behaviors such as having sex without proper consent and without thoroughly screening potential clients, which has been linked to an increase in professional violence and hostility toward them [21]. As a result, the authors noted certain study gaps. Upon reviewing the literature, the authors did note that there is not much qualitative and quantitative research that concentrates on the experiences of caregivers or parents who work in the sex industry. In order to better guide the creation of social policies and services that are supportive of sex workers and specifically cater to their needs, this study aims to examine the experiences and difficulties faced by female sex workers who also hold the role of caregiver or parent.

## 2. Literature Review Methodology

A literature review is a thorough examination of previous studies that are relevant to a given subject [22]. Through a critical evaluation and synthesis of the existing literature, it offers a contextual understanding [22]. The methodology of a scoping review was selected in order to synthesize the existing literature on the experiences of female sex workers who are also parents by identifying important research themes and gaps in the context of the literature. To accomplish the goal of the study, the authors used the five steps of the Arksey and O’Malley framework [23]: (1) identifying the research question; (2) identifying the relevant studies; (3) study selection; (4) charting the data; and (5) collating, summarizing, and reporting results [23].

### 2.1. Research Question

The following research questions guided this study:

- What are the common experiences, challenges, and support needs of female sex workers who are parents or caregivers?
- What gaps exist in the current literature regarding the intersection of sex work and parenthood, and what areas require further exploration or investigation?

### 2.2. Identifying Relevant Studies and Studies Selection

**Figure 1:**
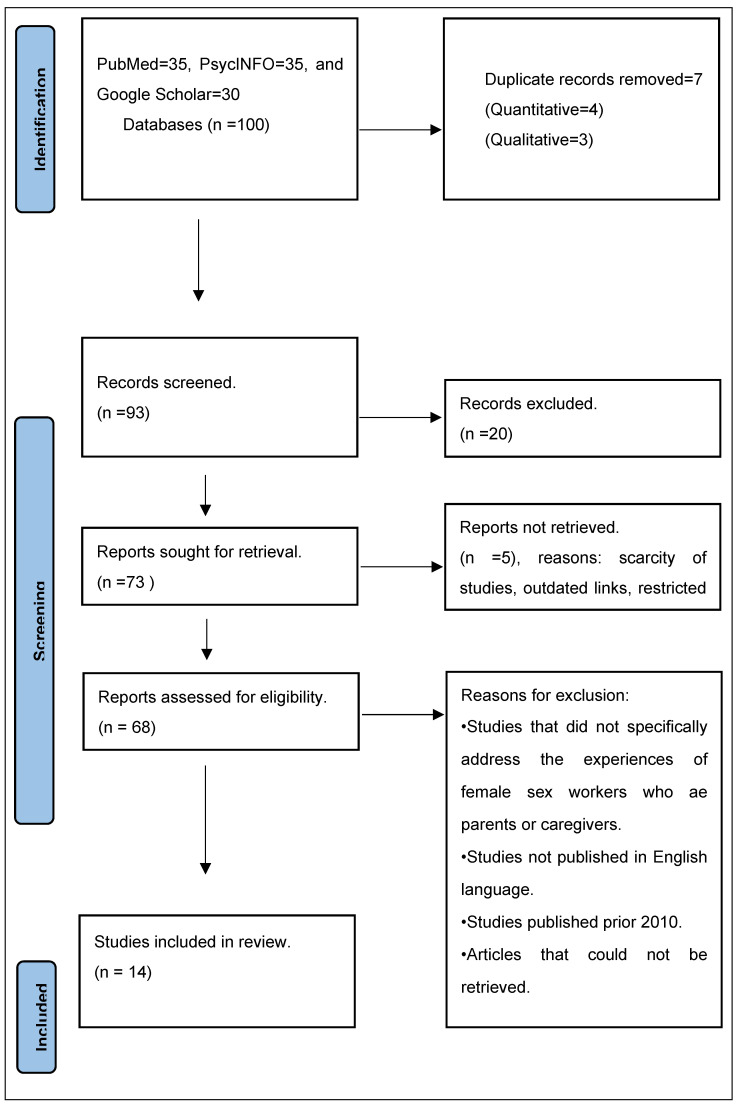
Flow chart representing the search strategy [24].

The authors conducted a search strategy in January 2024. The search terms were conducted across electronic databases such as PubMed, PsycINFO, and Google Scholar. The search strategy was developed in consultation with the team. The strategy aimed to locate both published and unpublished primary studies and reviews. A comprehensive search method was developed using the index terms used to define the publication, as well as the text words provided in the title and abstracts of relevant articles. The full search strategies are provided in Appendix A. More research that might have qualified for this review’s inclusion was found by manually searching the reference list of eligible studies. The search keywords had to be written in English and published no longer than twenty years ago. The authors identified 100 studies from electronic databases. Only five studies were not retrieved because of outdated links and the unavailability of studies in digital archives. Then, 14 relevant studies met the inclusion criteria. The selection process for studies is depicted in Figure 1. For eligibility criteria, the inclusion and exclusion criteria are explained as follows:

#### 2.2.1. Inclusion Criteria

- Studies published in English between 2010–2023.
- Studies focused on the experiences, challenges, and support needs of female sex workers who are parents or caregivers.

#### 2.2.2. Exclusion Criteria

- Studies that did not specifically address the experiences of female sex workers who are parents or caregivers.
- Studies not published in the English language.
- Studies published prior to 2010.
- Articles that could not be retrieved.

### 2.3. Data Extraction and Data Analysis

Using a piloted, tailored data extraction sheet, the two authors (L.W. and G.O.) and one invited reviewer independently extracted the data. The following titles were used to extract the data: authors, publication year, study nation, age range, sample size, and gender of participants. Disagreements between the reviewer and writers were settled through conversation. By grouping the research together and creating codes, the authors were able to use Creswell’s Tesch Method of data analysis to ultimately formulate the primary themes of this review [25].

## 3. Results

Out of this review, 14 pertinent studies were found. The plurality of research was qualitative. The features of the chosen articles are compiled in Table 1. Table 1 presents data indicating that the majority of the publications (n = 78.57%) incorporated in this analysis were from research done mostly in India, Mexico, and the UK and published between 2010 and 2015. There was only one systematic review included in this study. More study projects employed cross-sectional, qualitative, and quantitative research approaches. Moreover, all of the participants were female prostitutes. Thus, five major themes emerged from this study: (1) social stigma and healthcare discrimination; (2) legal challenges; (3) mental nexus; (4) risk behaviors and exposing children to a hazardous environment; and (5) social support.

### 3.1. Charting of Data

In this section, the authors drew a table that summarizes the characteristics of the articles selected in this review. Table 1 clarifies the features of the studies in terms of the year of publication, the country of study, the methodologies employed, and the analysis used.

### 3.2. Collating, Summarizing, and Reporting the Results

The authors again tabulated critical information from the included studies and quantified their characteristics. They then synthesized the research findings thematically. Five themes were developed, which were agreed upon by the authors and the reviewer. Table 2 shows the list of included studies, which include the following titles: authors, year of publication, country of study, age range, sample size, and participant gender.

#### 3.2.1. Social Stigma and Healthcare Discrimination

Female sex workers who are parents face profound stigma and discrimination that permeates many facets of their lives, largely due to societal prejudices [2,4,5]. Social stigma often manifests through the negative stereotypes society holds about sex workers, viewing them as morally unfit or irresponsible [2,4,5]. This perception can lead to significant social isolation, as sex worker parents may be excluded from community activities, judged harshly by other parents, and ostracized by community members [26]. If the job of their parent is found out, children of sex workers may be the target of bullying and discrimination, which could negatively affect their mental health and academic achievement [5,26]. Reducing their involvement in their children’s education and school activities is another way that parents of sex workers may feel judged or rejected by other parents and school staff [27]. Furthermore, the children of female sex workers in Kenya had to drop out of school due to poverty, and in India, female sex workers experienced challenges in having their children admitted to schools [27]. Discrimination in healthcare is another important problem. Healthcare professionals who are prejudiced against sex workers frequently criticize them and give them poor treatment [26]. Their children may likewise be subjected to prejudice as a result of this discrimination. Consequently, parents of sex workers may choose not to seek medical attention for their children or themselves, which could have a negative impact on their health [26].

#### 3.2.2. Legal Challenges

Many nations have legalized sex work. Parents who are sex workers face even more hurdles due to legal issues. Sex work involvement can be used against a parent in child custody hearings in several jurisdictions, where the court may examine the parent’s suitability to parent based only on their career [18]. Many parents of sex workers are forced by this legal action to conceal their employment, which adds to their stress and makes it more difficult for them to get the help they need [18]. Female sex workers may give up custody of their child to social services, the child’s father, friends, or other family members. In sub-Saharan Africa, child fostering was a common practice for the offspring of sex workers, and female sex workers in Uganda used this as a way to keep their children away from their jobs [28]. Female sex workers (FSWs) from affluent nations such as the United States, the United Kingdom, Canada, Mexico, and Singapore were more likely to say that their drug use, sex work, violence, homelessness, incarceration, and other disadvantaged lifestyles had driven them to give up their children [5,13]. None of the female sex workers who reared their children in India’s red-light district, however, claimed to have lost custody of their children [5,13].

**Table 2:** Summary of included studies.

Author/Year	Country/Location	Study Design	Sample Size (n)	Age Range (years)	Gender of Participants	Outcomes/Findings
Nestadt et al. 2021 [1].	U.S.A	Quantitative	214	≥13	Female sex workers	FSWs who are engaged in sex work in order to support their children are distinct from their counterparts in some key areas related to HIV and other health outcomes, including protective behaviors related to drug use and sex work and greater concerns about mental health.
Dodsworth (2014) [5].	UK	Qualitative	24	14–31	Female sex workers	The findings indicate that there is a need to cope with the threat to identity inherent in society’s diametrically opposed perceptions of the sex worker and the “good mother” and, simultaneously, to manage the coexistence of the roles and identities of mother and sex worker.
Zalwango et al. 2011 [6].	Uganda	Qualitative	100	≥20	Female sex workers	The money women got selling sex, and from other jobs they did, helped some to independently pay for their children’s school fees, their house rent, and food for their family without receiving support from partners.
Beckham et al. 2015 [8]	Tanzania	Qualitative	22	≥18	Female sex workers	Sex workers/mothers were aware of risks at work, but with children to support, their choices were constrained.
Rolon et al. 2013 [9]	Mexico	Mixed method	428	≥18	Female sex workers	Childrearing costs motivated sex work and structural constraints prevented couples from enacting lifestyle changes.
Basu and Dutta (2011) [10].	India	Qualitative	46	≥18	Femele sex workers	Sex workers who saw themselves as “mothers first” influenced localized patterns of HIV/AIDS communication and related work practices.
Papworth et al. 2015 [11].	Burkina Faso	Cross-sectional	533	≥18	Female sex workers	Motherhood was predictive of reporting limited difficulty when accessing health services.
Servin et al. 2017 [12]	Mexico	Cross-sectional	603	≥18	Female sex workers	This study highlights the high proportion of FSWs who are mothers and are financially responsible for their household.
Duff et al. 2015 [13].	Canada	Cross-sectional	399	≥14	Female sex workers	Findings highlight a critical need for tailored and nonjudgmental services and supports, including improved programs to address intersecting aspects of poverty, health literacy, stigma, and substance use.
Elsdon et al. 2021 [17]	UK	Qualitative	6	≥18	Female sex workers	Experiences reflected the diversity of bonding experiences held within the general population; they were also significantly influenced by contextual factors surrounding their work.
Duff et al. 2014 [20].	Canada	Quantitative	350	≥14	Female sex workers	The prevalence of child apprehension among mothers was 38.3%, with 37.4% reporting having been apprehended themselves by child welfare services.
Pravin, and Jogdand (2012) [27].	Asia, India	Quantitative	87	21–39	Female sex workers	Commercial sex workers conceived and decided to have a child under different circumstances. A few had children due to ignorance about contraceptives and MTP, but many decided to continue the pregnancy, as they wanted children.
Ma et al. 2019 [29].	Systematic review	Systematic review	21	≥18	Female sex workers	There are conflicting identities between sex work and motherhood and responses to social expectations of ideal motherhood.
Willis et al. 2014 [30].	Bangladesh	Qualitative	33	≥18	Female sex workers	Findings indicate that stigmatization of and discrimination against these children and their mothers are underlying conditions that compromise their access to safe housing, childcare, healthcare, education, and the protection of law enforcement.
Reed et al. 2013 [31].	India	Quantitative	850	≥18	Female sex workers	Findings suggest that challenging responsibilities related to the caretaking of children are associated with heightened vulnerability of HIV risk among FSWs.

In Canada, 38.3% of female sex workers experienced child custody loss, and almost 30% needed counseling to deal with the trauma of that experience [13]. For fear of losing custody of their children, over 13% of female sex workers refrained from contacting social services [13]. In certain underdeveloped nations, mothers who worked as sex workers had custody of their children, but there was a chance that they were putting them in danger. For instance, around 35% of FSWs’ children in India were raised in brothels [27].

#### 3.2.3. Mental Nexus

Sex worker parents’ mental health might be negatively impacted by their ongoing worry and anxiety about being judged [13]. High levels of anxiety, depression, and other mental health problems are a result of persistent prejudice and social pressure [26]. Their inability to parent effectively may be impacted by this mental health load, which could lead to difficult circumstances for both the parents and their children [26]. Additionally, some people turn to drugs as a coping technique due to the stress of dealing with stigma, financial instability, and the demands of their multiple roles [27]. This use can increase shame and anxiety, especially when it comes to their capacity to give their children a safe and healthy environment. Their ability to parent may also be hampered by drug and alcohol abuse, which could result in inconsistent or neglectful caring [26].

#### 3.2.4. Risk Behaviors and Exposing Children to a Hazardous Environment

Some female sex workers’ parenting approaches and talents were negatively impacted by their marginalized lifestyles and workplace hazards. In addition to increasing the likelihood that they will suffer a miscarriage or stillbirth and have congenital birth defects in their offspring, a number of risks, including STIs, addiction, violence, malnutrition, and inadequate prenatal care, may also make their children more susceptible to violence, sex abuse, sexual activity, drug addiction, and involvement in the sex industry [28,29,30]. Quantitative research findings generally observed that motherhood significantly influences the choice of sex work, work practices, and access to social services and healthcare for female sex workers [11,13]. According to an Indian study, female sex workers were more likely to demand more payment for unprotected sex and to report inconsistent condom use if they had three or more children or children with health issues [31]. Some female sex workers were wary of engaging in risky activities related to HIV in order to preserve their health for their offspring. Maternity was linked to fewer reports of unprotected anal or vaginal intercourse with new clients in Burkina Faso, as well as fewer difficulties obtaining healthcare [11]. Seventy-five percent of Kenya’s female prostitutes operated out of their homes [29].

#### 3.2.5. Social Support

Social support networks were essential to maintaining FSWs’ ability to carry out their roles as mothers. For instance, non-governmental organizations (NGOs) in Singapore and India provide training and resources to female sex workers, including food, clothes, secure housing, residential schools for their children, outreach to the children of FSWs, and skill development [10,27,29]. The stress and load of raising children were greatly reduced for FSWs because of this outside social assistance, which also gave them chances to preserve and uphold their identity as mothers [29].

## 4. Discussion

This scoping review aimed to review the experiences and challenges of sex workers who are parents or caregivers. The selected time frame for this scoping review, covering articles from 2010 to 2023, was chosen to ensure the inclusion of the most recent and relevant research on the experiences of female sex workers who are also parents or caregivers. This period captures significant changes in societal attitudes, legal frameworks, and support systems related to sex work and parenthood. This discussion also delves into the implications of these findings and underscores the importance of further research and supportive policies in this area of study. This study revealed that sex workers who are parents or caregivers must balance the demands of their profession with the responsibilities of raising and caring for children, often in environments characterized by stigma, discrimination, and legal vulnerabilities. This study also revealed the pervasive influence of societal attitudes and structural inequalities on the wellbeing of sex worker parents and their families [18]. Stigma and discrimination not only impact the mental health and social integration of sex workers but also pose risks to the safety and stability of their children [26]. The lack of legal protections and access to essential services further exacerbates the vulnerabilities faced by this population, leaving it at a heightened risk of exploitation and marginalization [26].

The legal context of sex work significantly impacts the policies and support systems available to sex workers and their children. Notably, in countries where sex work is legal and regulated, sex workers are more likely to have access to labor rights, healthcare, and social services, which can provide a more stable environment for their children [32]. Legalization often comes with the implementation of health and safety standards, reducing the stigma and discrimination faced by sex workers, which can positively influence their parenting experiences [32]. Conversely, in countries where sex work is illegal, sex workers operate in a more precarious and marginalized context. This illegality often limits their access to essential services and exposes them to higher risks of violence, exploitation, and legal repercussions [33]. The lack of legal protection exacerbates the challenges they face in ensuring the wellbeing of their children. Policies in such contexts may be punitive rather than supportive, focusing on criminalization rather than providing necessary social services [33]. This environment can lead to increased stress, instability, and potential separation from their children due to legal interventions.

To address the key themes identified in the review, policymakers and legislators should work towards decriminalizing and regulating sex work to provide legal protections and access to essential services for sex workers and their children. Healthcare providers need to develop targeted health programs, including mental health support and reproductive health services, in a non-judgmental environment. Social services and child welfare agencies should create specialized programs offering affordable childcare, parenting education, financial assistance, and housing support. Community organizations and non-governmental organizations (NGOs) should focus on advocacy, legal assistance, and reducing stigma, while researchers should continue to explore the intersection of sex work and parenthood to provide evidence-based recommendations for policy and practice.

However, this study discovered several limitations. Firstly, the scope of this review is limited to the studies included in the analysis, which may not fully capture the detailed experiences of sex worker parents if there are biases in the existing literature. Additionally, publication bias may influence the review’s conclusions, as studies with significant findings may be more readily published, potentially skewing the representation of certain experiences. The heterogeneity of study designs, populations, and conceptual frameworks among the included studies could also impact the generalizability of the review’s findings. Language and geographic biases may further limit the review, as studies published in languages other than English or conducted in certain geographic regions may have been overlooked. Finally, temporal limitations, based on the cutoff date for included studies, may omit more recent research and fail to reflect emerging trends or policy changes in sex work and parenthood.

## 5. Conclusions

In conclusion, this scoping review provides valuable insights into the experiences of sex workers who are also caregivers or parents, highlighting the urgent need for further research and targeted interventions in this field. By amplifying the voices of this marginalized population and advocating for their rights and wellbeing, we can strive towards a more inclusive and equitable society for all individuals, regardless of their occupation or parental status.

## Figures and Tables

**Table 1:** Summary characteristics of included studies.

Year of Publication (n= 14)	Frequency	Percentage
2010–2015	**11**	78.57%
2016–2020	2	14.29%
2021–2024	1	7.14%
**Country (n = 14)**	**Frequency**	**Percentage**
USA	**1**	7.14%
UK	2	14.29%
Uganda	1	7.14%
Tanzania	1	7.14%
Mexico	2	14.29%
India	3	21.43%
Burkina Faso	1	7.14%
Canada	2	14.29%
Bangladesh	1	7.14%
**Methodology Used (n = 14)**	**Frequency**	**Percentage**
Qualitative	**5**	35.71%
Quantitative	4	28.60%
Cross-sectional	3	21.43%
Systematic review	1	7.14%
Mixed method	1	7.14%

## Data Availability

No new data were created or analyzed in this study. Data sharing is not applicable to this research.

## Appendix A. Full Search Strategies for Each Database

**Database**	**Search Strategy**
***PubMed***	1. *(“sex workers” OR ‘prostitutes” OR “sex trade workers” OR “commercial sex workers” OR “sex industry workers”) AND (“parenting” OR “caregiving” OR “challenges” OR “support needs”) [Title/Abstract]* 2. *(“sex workers” OR “prostitutes” OR “sex trade workers” OR “commercial sex workers” OR “sex industry workers”) AND (“parent*” OR motherhood” OR “fatherhood” OR “child rearing”) AND (“experiences” OR “challenges” OR “support needs”) [Title/Abstract]* 3. *(“sex work” OR “prostitution” OR “sex trade” or “commercial sex work” OR “sex industry”) AND (“parenting” OR “caregiving” OR “parenthood”) AND (“experiences” OR “challenges” OR “support needs”) [Title/Abstract]*
***PsycINFO***	1. *(AB (“sex workers” OR “prostitutes’ OR “sex trade workers” OR “commercial sex workers” OR “sex industry workers”)) AND (AB (“parenting” OR “caregiving” OR “parenthood’)) AND (AB (“experiences” OR “challenges” OR “support needs”)) [Title/Abstract]* 2. *(TI (“sex work” OR “prostitution” OR “sex trade” OR “commercial sex work” OR “sex industry”)) AND (“parenting” OR “caregiving” OR “parenthood)) AND (AB (“experiences” OR “challenges” OR “support needs”) [Title/Abstract]*
***Google scholar***	1. *(“sex workers” OR “prostitutes’ OR “sex trade workers” OR “commercial sex workers” OR “sex industry workers”)) AND (“parenting” OR “caregiving” OR “parenthood’) AND (“experiences” OR “challenges” OR “support needs”) [Title/Abstract]* 2. *(“sex work” OR “prostitution” OR “sex trade” OR “commercial sex work” OR “sex industry”) AND (“parenting” OR “caregiving” OR “parenthood) AND (“experiences” OR “challenges” OR “support needs”) [Title]*
